# The impact of narcissistic rivalry on work burnout among working adults: the mediating effects of perceived stress and sleep health

**DOI:** 10.3389/fpsyg.2026.1759736

**Published:** 2026-04-21

**Authors:** Weixian Wang, Yue Zhao, Yanan Ruan, Luyao Xiong, Tingting Bai, Hui Zhao, Yun Yun, Fuzhi Wang

**Affiliations:** 1 School of Nursing, Bengbu Medical University, Bengbu, China; 2 School of Medical Information and Engineering, Bengbu Medical University, Bengbu, China

**Keywords:** employed individuals, job burnout, narcissistic rivalry, perceived stress, sleep health

## Abstract

**Objective:**

To evaluate the association between narcissistic rivalry and work burnout among employed adults aged 24–60 years in mainland China and to examine whether perceived stress and sleep health show indirect effects in this relationship.

**Methods:**

Employed individuals aged 24–60 years from the 2024 Chinese Residents’ Psychological and Behavioral Survey were included in this study. Data were collected using the General Information Questionnaire, Narcissistic Admiration and Rivalry Questionnaire Short Scale (NARQS), Perceived Stress Scale (PSS-4), Sleep Health Self-Assessment (SHS), and Chinese Copenhagen Burnout Inventory (CCBI). Spearman’s correlation analysis was used to examine the relationships among narcissistic rivalry, perceived stress, sleep health, and work burnout. Mediation analysis was conducted to explore the mediating roles of perceived stress and sleep health in the relationship between narcissistic rivalry and work burnout. In addition, age-stratified analyses were performed to explore whether the mediation pattern varied across age groups.

**Results:**

Narcissistic rivalry was positively correlated with work burnout (*r_s_* = 0.362, *p* < 0.01). Work burnout was also positively correlated with perceived stress (*r_s_* = 0.530, *p* < 0.01) and negatively correlated with sleep health (*r_s_* = −0.212, p < 0.01). Mediation analysis showed that narcissistic rivalry had a significant direct effect on work burnout (95% CI: 0.327–0.496), accounting for 57.81% of the total effect, and a significant indirect effect through perceived stress and sleep health (95% CI: 0.239–0.365), accounting for 42.19% of the total effect. In age-stratified analyses, all three indirect pathways were significant in the young group, whereas in the middle-aged group the indirect pathway through sleep health alone was not significant, suggesting that the pattern of indirect effects may differ across age groups.

**Conclusion:**

Narcissistic rivalry was directly associated with work burnout, and perceived stress and sleep health showed significant indirect effects in this association. The mediation pattern was broadly similar across age groups, although the pathway through sleep health alone appeared weaker in middle-aged employed adults.

## 1 Introduction

China is presently experiencing a pivotal phase of societal transition and rapid development. The online terms “lying flat” and “involution” reflect the helplessness and challenges encountered by certain social groups when coping with work responsibilities amid escalating competition and increasing psychological stress, and these phenomena have drawn growing attention to work-related burnout (Zhongting and Yan, 2023). Work burnout refers to adverse emotional reactions to one’s work. This condition not only reduces job satisfaction and increases turnover intentions (Üngüren et al., 2024), but may also negatively affect colleagues, intensify interpersonal conflict, disrupt work performance, and influence the workplace atmosphere (Almeida et al., 2023).

Comprehensive studies have highlighted a notable relationship between burnout and personal characteristics. Sociable individuals who enjoy interpersonal interactions, demonstrate cooperation and friendliness, and possess empathy tend to report lower levels of burnout. In contrast, those who exhibit emotional instability, hostility, suspicion, and a lack of cooperativeness tend to report higher levels of burnout (Angelini, 2023). An individual’s personality traits are frequently associated with his or her personality features, particularly narcissistic qualities (Allport, 1937). Narcissism is a self-focused psychological characteristic defined by a strong preoccupation with self-image and self-esteem, a tendency to seek external affirmation and admiration, and a pronounced sense of distinctiveness (Yutong, 2024). Narcissism comprises two dimensions: narcissistic admiration and narcissistic rivalry. Narcissistic admiration is typically associated with confidence, extroversion, humor, and charm, whereas narcissistic rivalry is more closely related to impulsivity, aggression, selfishness, and arrogance (Back et al., 2013). Narcissistic rivalry is characterized by an inflated yet vulnerable self-image, in which individuals seek to become the focus of attention and long for recognition from others. However, this egocentric tendency may fail to produce the desired interpersonal outcomes and may ultimately contribute to relationship breakdowns (Seidman and Schlott, 2022), which may in turn be linked to work-related burnout through social isolation (Jinjin and Bo, 2019). Consistent with this possibility, Rana et al. (2022) found in their investigation of surgeons’ narcissism and burnout that narcissistic admiration may serve as a protective factor against burnout, whereas narcissistic rivalry was associated with higher levels of burnout. A possible explanation is that individuals with narcissistic rivalry tend to engage in upward social comparison in order to preserve a sense of superiority (Helgeson and Mickelson, 1995). Although the association between narcissistic rivalry and burnout has received increasing attention, the extent to which additional factors may be involved in this relationship remains unclear.

In addition to personality factors, the roles of stress and sleep problems in work burnout have also received increasing attention. Stress is a dynamic psychological process in which an individual’s response to stressors is shaped by cognitive appraisal, personality attributes, social support, and coping strategies (Ringwald et al., 2024). Perceived stress, a key component of stress, refers to an individual’s evaluation of stressors in his or her environment (Schneider, 2022). Among employed people, stress levels are positively associated with burnout severity (Si̇s Çeli̇k and Kılınç, 2022). However, focusing exclusively on the association between stress and burnout may be insufficient for understanding how burnout develops. Previous studies have suggested that stress may be linked to work burnout through sleep-related processes (Zarei and Fooladvand, 2022). Prolonged stress may induce abnormal psychological states and disrupt physiological rhythms. As an important link between psychological functioning and physical health, poor sleep may be involved in the association between stress and burnout (Song et al., 2020).

Overall, many researchers have examined the roles of personality traits, perceived stress, and sleep-related problems in work burnout. However, in mainland China, the association between narcissistic rivalry and work burnout, as well as the potential roles of perceived stress and sleep health in this association, has not been sufficiently examined. In addition, these associations may differ across age groups. Individuals in different age groups tend to assume different social roles, which may be associated with age-specific manifestations of narcissistic traits and differences in work burnout risk (Weidmann et al., 2023; Mendes and Miguel, 2024). Previous research has also indicated that work burnout may peak around the age of 45 years (Yang et al., 2017). Therefore, examining age-group differences may help clarify whether the associations among narcissistic rivalry, perceived stress, sleep health, and work burnout differ between younger and middle-aged employed adults.

Against this background, the present study aimed to examine the association between narcissistic rivalry and work burnout among employed adults in mainland China and to test whether perceived stress and sleep health showed indirect effects in this association. Furthermore, age-stratified analyses were conducted to explore whether these indirect effects differed between younger and middle-aged groups, which may provide further insight into factors associated with work burnout and inform age-sensitive intervention strategies for employed adults.

## 2 Data and methods

### 2.1 Data source and sample

The 2024 Chinese Residents’ Psychological and Behavioral Survey provided the data for this study. The analysis included provincial capitals and municipalities directly managed by the central government in all Chinese provinces and autonomous regions except for Taiwan. There were 150 non-capital cities from each province and autonomous district selected using random number tables. Stratified quota sampling was conducted with reference to the 2020 Seventh National Population Census, and convenience sampling was used within each quota. The Ethics Review Committee of Shanghai Jiao Tong University approved this study (H20240237I).

Participants were eligible if they were Chinese residents, provided informed consent, and were able to complete the questionnaire independently or with assistance. For the present analysis, we included employed adults aged 24–60 years. Students, unemployed individuals, those without stable employment, and retirees were excluded, as were respondents younger than 24 or older than 60 years. The final sample comprised 1,297 participants.

### 2.2 Survey tools

#### 2.2.1 General information questionnaire

The General Information Questionnaire collects data concerning gender, age, employment status, educational attainment, marital status, per capita monthly household income, family structure, and personal health condition.

#### 2.2.2 Short version of the narcissistic admiration and rivalry questionnaire short scale (NARQS)

This scale was developed by Back et al. (2013) and adapted for Chinese populations by Deng et al. (2017). The revised version of the NARQS includes two dimensions: narcissistic admiration and narcissistic rivalry. In this study, the narcissistic rivalry dimension was used as the survey tool. The tool employs a six-point Likert scale that ranges from 1 = strongly disagree to 6 = strongly agree. The Cronbach’s *α* coefficient of the scale is 0.828.

#### 2.2.3 Perceived stress scale (PSS-4)

The Chinese Perceived Stress Scale (PSS-4) was utilized to evaluate people’s reported stress throughout the preceding month (Cohen, 1988). The PSS-4 is the most succinct iteration of the PSS and is ideal for convenience surveys. The assessment has four items, and each are evaluated on a five-point Likert scale from 1 (“Never”) to 5 (“Very often”). Scores range from 4 to 20, with higher scores indicating greater perceived stress (Du et al., 2023). The Cronbach’s α coefficient for this scale is 0.919.

#### 2.2.4 Sleep health self-assessment (SHS)

The SHS, developed by Fan et al. (2020), integrates five sleep factors—sleep duration, chronotype, snoring, daytime drowsiness, and insomnia—to produce a comprehensive sleep health score. The overall score varies from 0 to 5, with higher scores signifying healthier sleep patterns. Although the SHS is a brief 0–5 composite indicator, it integrates several core dimensions of sleep health, including sleep duration, chronotype, snoring, daytime drowsiness, and insomnia. Consistent with previous research, the present study used the SHS to assess sleep health in a large-scale survey context. Its brevity makes it suitable for such research by reducing respondent burden (Goodman et al., 2025).

#### 2.2.5 Chinese Copenhagen burnout inventory (CCBI)

The CCBI was developed by Kristensen et al. (2005). In this study, the work burnout subscale was used to assess the extent of the physical and mental fatigue experienced by individuals in the workplace. The CCBI (Yeh et al., 2007) divides the work burnout subscale into two dimensions, namely, “work exhaustion” and “work frustration,” and consists of a total of 7 items. A five-point Likert scale was used, with higher scores indicating higher levels of burnout. The Cronbach’s *α* coefficient of the scale is 0.756.

### 2.3 Quality control

The questionnaire was developed through expert consultation and pilot testing before formal implementation. All investigators received standardized training before data collection. Questionnaires were administered electronically, and the data were checked regularly during the survey process. After data collection, two independent reviewers conducted logical checks and data screening, and any outliers were verified before analysis.

### 2.4 Statistical analysis

A statistical analysis was performed using SPSS 27.0. Harman’s single-factor test was performed to assess common method bias (Podsakoff et al., 2003). Continuous variables are presented as medians and interquartile ranges (IQRs), and categorical variables are presented as *n* (%). Spearman’s correlation analysis was used to examine the variable relationships. Model 6 from Hayes’ SPSS Process macro was used for mediation analysis (Hayes, 2017), and Origin 2021 was employed for data visualization.

## 3 Results

### 3.1 Common method bias test

All variables in this study were measured using self-report scales. To examine the potential issue of common method bias, Harman’s single-factor test was conducted. The results showed that the variance explained by the first principal factor was 31.50%, which was below the 40% threshold, indicating that no significant common method bias was present in this study.

### 3.2 General information

This study included 1,297 participants. The demographic characteristics of the survey sample are presented in Table 1.

**Table 1:** General characteristics of the study participants (*n* = 1,297).

Project		Number of participants (percentage, %)
Gender	Male	706 (54.4%)
	Female	591 (45.6%)
Age	24–44	862 (66.5%)
	45–60	435 (33.5%)
Religious belief	None	1,224 (94.4%)
	Yes	73 (5.6%)
Marital status	Unmarried	234 (18.0%)
	Married	1,034 (79.7%)
	Divorced	21 (1.6%)
	Widowed	8 (0.6%)
Education	Junior high school or below	254 (19.6%)
	High school/Vocational school	241 (18.5%)
	College/Undergraduate	726 (56.0%)
	Master’s degree or above	76 (5.9%)
Ethnicity	Han ethnicity	1,285 (99.1%)
	Ethnic minorities	12 (0.9%)
Per capita monthly household income	<4,000	444 (34.2%)
	4,000~	584 (45.0%)
	>9,000	269 (20.7%)

The narcissistic rivalry, perceived stress, sleep health, and work burnout scores of the study participants are presented in Table 2.

**Table 2:** Participants’ scores on each variable [*n* = 1,297, Scores, presented as median (IQR)].

Item	Dimension	Total score
Narcissism		20 (16, 23)
	Narcissistic rivalry	9 (6, 11)
	Narcissistic admiration	11 (9, 13)
Work burnout		19 (14, 22)
	Work frustration	11 (8, 12)
	Work exhaustion	9 (6, 10)
Perceived stress		8 (4, 10)
Sleep health		3 (2, 4)

### 3.3 Correlation analysis of narcissistic rivalry, perceived stress, sleep health, and work burnout among the research participants

**Figure 1:**
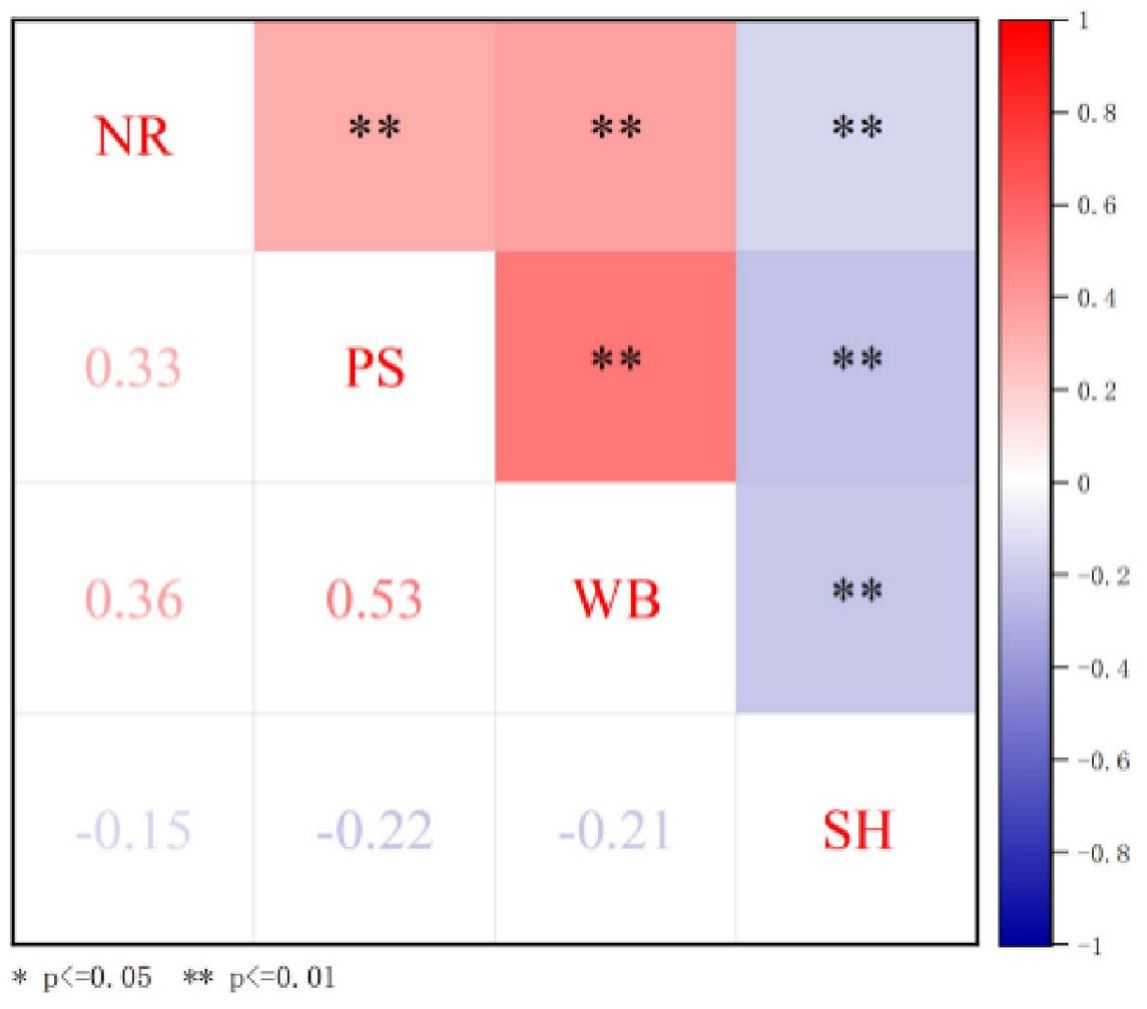
Heatmap depicting the correlations among all variables in the overall sample.

Spearman’s correlation was used to analyze the correlations among the study variables. The results revealed a negative correlation between narcissistic rivalry and sleep health (*r_s_* = −0.147, *p* < 0.01) and positive correlations with perceived stress and work burnout (*r_s_* = 0.332, *p* < 0.01; *r_s_* = 0.362, *p* < 0.01). Work burnout was correlated positively with perceived stress (*r_s_* = 0.530, *p* < 0.01) and negatively with sleep health (*r_s_* = −0.212, *p* < 0.01); perceived stress also exhibited a negative correlation with sleep health (*r_s_* = −0.220, *p* < 0.01), as illustrated in Figure 1.

### 3.4 Chain mediating analysis of narcissistic rivalry, perceived stress, sleep health, and work burnout

#### 3.4.1 Chain mediating effect analysis of perceived stress and sleep health in the overall sample

Model 6 from Hayes’ SPSS Process plugin was employed in this study to examine the chain mediating effects. Narcissistic rivalry was identified as the independent variable (*X*), work burnout as the dependent variable (*Y*), and perceived stress and sleep health as the mediating variables (*M1*, *M2*). The control variables in the mediation study included gender, monthly family income per capita, and education.

A regression analysis was used to examine the mediating roles of perceived stress and sleep health in the relationship between narcissistic rivalry and work burnout, and the results are presented in Table 3. The regression equation of the whole model was statistically significant. Narcissistic rivalry was significantly and positively associated with perceived stress (*β* = 0.382, *p* < 0.001). Higher levels of narcissistic rivalry (*β* = −0.018, *p* < 0.05) and perceived stress (*β* = −0.052, *p* < 0.001) were associated with poorer sleep health. Narcissistic rivalry (*β* = 0.411, *p* < 0.001) and perceived stress (*β* = 0.734, *p* < 0.001) were positively associated with work burnout, whereas sleep health was a significant negative predictor of work burnout (*β* = −0.528, *p* < 0.001).

**Table 3:** Regression analysis of the overall mediation model.

Variable	Perceived stress		Sleep health		Work burnout	
	*β*	*t*	*β*	*t*	*β*	*t*
Gender	0.502	2.808^**^	0.006	0.120	0.630	2.370^*^
Monthly per capita household income	−0.038	−0.884	−0.007	−0.590	0.025	0.401
Educational attainment	−0.086	−1.794	−0.034	−2.436^*^	−0.231	−3.213^***^
Narcissistic rivalry	0.382	14.159^***^	−0.018	−2.164^*^	0.411	9.550^***^
Perceived stress			−0.052	−6.545^***^	0.734	17.485^***^
Sleep health					−0.528	−3.655^***^
*R* ^2^	0.139		0.054		0.348	
*F*	52.032^***^		14.647^***^		114.783^***^	

*p < 0.05, **p < 0.01, ***p < 0.001; same as below.

**Figure 2:**
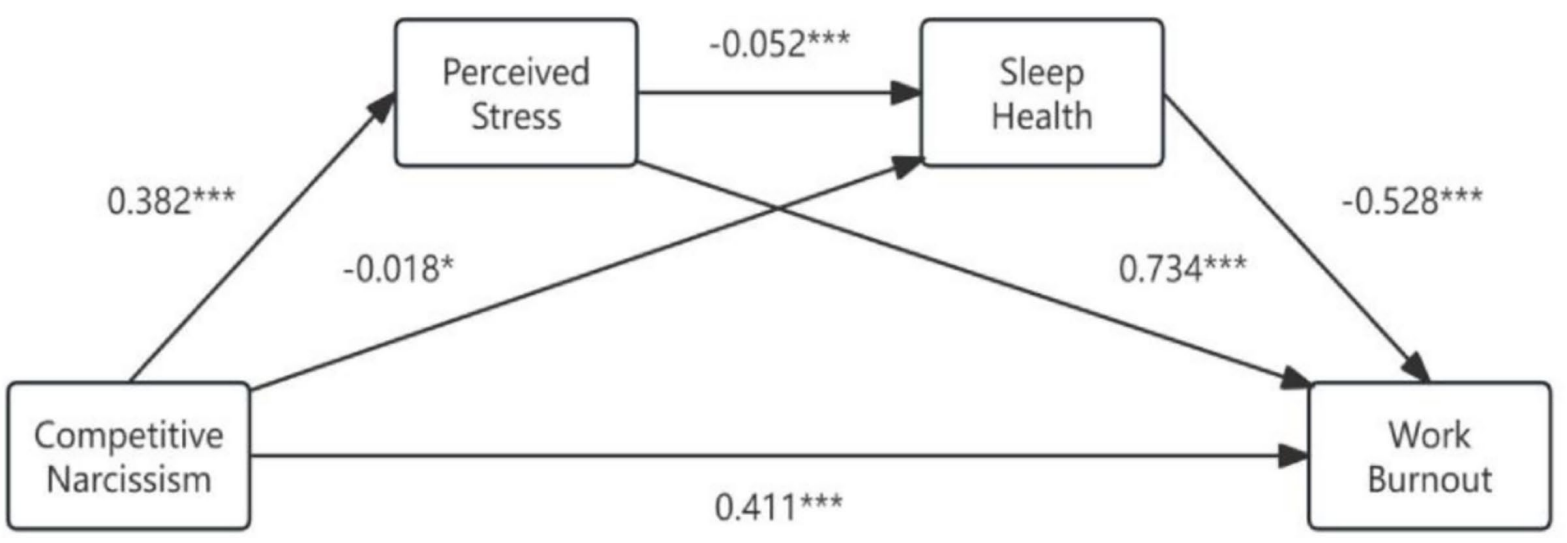
Overall chain mediating effect model.

Confidence intervals for the sample were computed with the bootstrap method. Model 6 from the SPSS Process plugin was selected, which utilized 5,000 consecutive repeated samples to derive 95% confidence intervals. The mediating effects of perceived stress and sleep health were significant, with the total indirect effect being 0.300, accounting for 42.19% of the total effect. The mediating impact consisted of three pathways: Ind1: Narcissistic Rivalry → Perceived Stress → Work Burnout (0.280); Ind2: Narcissistic Rivalry → Sleep Health → Work Burnout (0.010); and Ind3: Narcissistic Rivalry → Perceived Stress → Sleep Health → Work Burnout (0.011). The contributions of Ind1, Ind2 and Ind3 to the total effect were 39.37, 1.34, and 1.48%, with confidence intervals of [0.219, 0.346], [0.000, 0.022], and [0.004, 0.018], respectively, thus demonstrating that all three paths were statistically significant, as shown in Table 4. These findings suggest that the association between narcissistic rivalry and work burnout may involve perceived stress and sleep health. The overall chain mediating effect model is shown in Figure 2.

**Table 4:** Overall mediation decomposition table.

Effect	Path	Effect value	Effect contribution	Bootstrapped 95% confidence interval
Direct effect		0.411	57.81%	[0.327,0.496]
Mediating effect	Ind1	0.280	39.37%	[0.219,0.346]
	Ind2	0.010	1.34%	[0.000,0.022]
	Ind3	0.011	1.48%	[0.004,0.018]
	Total mediating effect	0.300	42.19%	[0.239,0.365]

#### 3.4.2 Chain mediating analysis of perceived stress and sleep health across different age groups

To further examine whether the indirect effects differed across age groups, the sample was divided into a young group and a middle-aged group, and subgroup analyses were conducted. The results revealed that in the young group, when narcissism rivalry was the predictor variable and perceived stress was the outcome variable, narcissistic rivalry was significantly and positively associated with perceived stress (*β* = 0.368, *p* < 0.001). When narcissistic rivalry and perceived stress were used as predictor variables and sleep health was used as the outcome variable, both narcissistic rivalry (*β* = −0.028, *p* < 0.01) and perceived stress (*β* = −0.042, *p* < 0.001) significantly negatively predicted sleep health. When narcissistic rivalry, perceived stress, and sleep health were used as predictor variables and work burnout was used as the outcome variable, both narcissistic rivalry (*β* = 0.378, *p* < 0.001) and perceived stress (*β* = 0.702, *p* < 0.001) significantly and positively predicted work burnout, whereas sleep health significantly negatively predicted work burnout (*β* = −0.448, *p* < 0.05). In the middle-aged group, when narcissistic rivalry was used as the predictor and perceived stress was used as the outcome variable, narcissistic rivalry showed a stronger positive association with perceived stress (*β* = 0.402, *p* < 0.001). When narcissistic rivalry and perceived stress were used as predictors and sleep health was used as the outcome variable, perceived stress was significantly and negatively associated with sleep health (*β* = −0.078, *p* < 0.001), whereas narcissistic rivalry was not significantly associated with sleep health (*β* = 0.006, *p* > 0.05). When narcissistic rivalry, perceived stress, and sleep health were included in the model, narcissistic rivalry (*β* = 0.486, *p* < 0.001) and perceived stress (*β* = 0.790, *p < 0*.001) were significantly positively associated with work burnout, while sleep health was significantly negatively associated with work burnout (*β* = −0.675, *p* < 0.01). These details are presented in Table 5.

**Table 5:** Regression analysis of the mediating models across age groups.

Group	Variable	Perceived stress		Sleep health		Work burnout	
		*β*	*t*	*β*	*t*	*β*	*t*
Young group	Gender	0.490	2.162^*^	−0.019	−0.299	0.465	1.377
	Monthly per capita household income	−0.012	−0.217	0.005	0.345	−0.007	−0.082
	Educational attainment	−0.064	−0.879	−0.003	−0.142	−0.200	−1.860
	Narcissistic rivalry	0.368	10.861^***^	−0.028	−2.762^**^	0.378	6.993^***^
	Perceived stress			−0.042	−4.500^***^	0.702	13.642^***^
	Sleep health					−0.448	−2.429^*^
	*R* ^2^	0.124		0.047		0.309	
	*F*	30.264^***^		8.383^***^		63.857^***^	
Midlife group	Gender	0.241	0.818	0.071	0.772	0.817	1.861
	Monthly per capita household income	−0.102	−1.463	−0.034	−1.545	0.090	0.868
	Educational attainment	−0.240	−3.263^**^	−0.051	−2.216^*^	−0.362	−3.253^**^
	Narcissistic rivalry	0.402	9.234^***^	0.006	0.421	0.486	6.860^***^
	Perceived stress			−0.078	−5.191^***^	0.790	10.687^***^
	Sleep health					−0.675	−2.931^**^
	*R* ^2^	0.193		0.078		0.439	
	*F*	25.665^***^		7.291^***^		55.776^***^	

The bootstrap results are shown in Table 6. In the young group, the total indirect effect was 0.277, accounting for 42.36% of the total effect. The effect values for the three indirect paths were 0.258 (39.40%), 0.012 (1.89%), and 0.007 (1.07%), with 95% confidence intervals not including 0, indicating significant indirect effects in the young group. In the middle-aged group, the total indirect effect was 0.335, accounting for 40.76% of the total effect. The effect values for the three indirect paths were 0.318 (38.69%), −0.004 (−0.51%), and 0.021 (2.58%). The 95% confidence intervals for Ind1 and Ind3 did not include 0, indicating statistical significance, while the 95% confidence interval for Ind2 included 0, indicating that this indirect path was not statistically significant. These findings suggest that, in the middle-aged group, no significant indirect effect through sleep health alone was observed in the association between narcissistic rivalry and work burnout.

**Table 6:** Decomposition of the mediating effects by age group.

Group	Path	Effect value	Effect proportion	Bootstrap 95% confidence interval
Young group	Direct effect	0.378	57.64%	[0.272, 0.484]
	Ind1	0.258	39.40%	[0.187, 0.335]
	Ind2	0.012	1.89%	[0.002, 0.028]
	Ind3	0.007	1.07%	[0.002, 0.015]
	Total mediation effect	0.277	42.36%	[0.205, 0.355]
Midlife group	Direct effect	0.486	59.24%	[0.347, 0.626]
	Ind1	0.318	38.69%	[0.210, 0.439]
	Ind2	−0.004	−0.51%	[−0.028, 0.022]
	Ind3	0.021	2.58%	[0.006, 0.042]
	Total mediation effect	0.335	40.76%	[0.228, 0.456]

## 4 Discussion

The employed adults in this study showed a moderate level of narcissistic rivalry, suggesting that competitive self-protective tendencies were present to some extent in this sample. This pattern may reflect a tendency for some employed adults to remain sensitive to social comparison and status-related threats in the workplace. The research data reveal that the subjects have moderate work burnout scores. Although a certain level of burnout is present, the overall level was not particularly high, suggesting that burnout symptoms among these employed adults were generally manageable. Furthermore, their reported stress levels were categorized as low to moderate, which may indicate that many participants retained a certain capacity to cope with challenges arising from work and daily life. Although the general sleep health of employed adults was moderate, more than half of the respondents reported experiencing insomnia symptoms, which likely indicates pervasive sleep quality concerns across the workforce.

A correlation analysis suggested that work burnout is positively correlated with narcissistic rivalry and perceived stress but is negatively correlated with sleep health. Falco et al. (2020) found that narcissistic traits were associated with a greater tendency toward workaholism, particularly under conditions of high workload, and workaholism has in turn been linked to work burnout. The present findings are consistent with this association and further suggest that when narcissistic rivalry is more pronounced—particularly when individuals are more preoccupied with maintaining a superior position in social comparisons—perceived stress may also be higher. Intense work-related stress associated with narcissistic rivalry was related to higher levels of emotional exhaustion, a key indicator of burnout (Edú-Valsania et al., 2022). In addition, this study found that poorer sleep health was associated with higher levels of work burnout, which is consistent with the systematic review by Demerouti (2024), indicating that prolonged exposure to stress and insufficient recovery may increase the risk of burnout.

A subsequent analysis of the mediation model indicated that perceived stress and sleep health showed partial indirect effects in the association between narcissistic rivalry and job burnout among employees in mainland China. Specifically, narcissistic rivalry was directly related to job burnout and also showed indirect links to job burnout through perceived stress and sleep health. Individuals with narcissistic rivalry tend to seek recognition and strive to be the center of attention to secure prospective support resources (Wright and Hobfoll, 2004); however, when confronted with concerns about social comparison and the possibility of being outperformed in important domains, they may be more likely to withdraw from interpersonal connections, which may make social acceptance more difficult (Nicholls and Stukas, 2011). This personality tendency may be associated with more negative workplace experiences (Greenbaum et al., 2024), poorer interpersonal relationships (Wehner and Ziegler, 2023), greater emotional distress (Richter et al., 2023), and reduced support (Yu et al., 2022). In turn, these characteristics may be related to a lower sense of competence and achievement, greater emotional exhaustion, and fewer personal resources, and thus to higher levels of burnout. Moreover, perceiving oneself as being surpassed by others may be associated with higher levels of perceived stress and poorer sleep (Mao et al., 2023). Persistent sleep problems may, in turn, be related to impaired recovery (Albakri et al., 2024), sustained stress (Meng et al., 2025), poorer work performance (Joshi and Patil, 2025), lower well-being, and higher levels of burnout. Notably, the indirect effect of narcissistic rivalry operating solely through sleep health was relatively small and clearly weaker than the indirect effect operating solely through perceived stress. This finding may indicate that sleep health is more closely intertwined with the stress process than functioning as a strong independent pathway in this model. At the same time, sleep health is inherently multifactorial. Adult sleep is influenced by a wide range of biological, behavioral, environmental, and socioeconomic factors (Philippens et al., 2022), and thus the independent contribution of a single personality-related factor to sleep-related outcomes may be relatively limited. Therefore, the relatively small sleep-related indirect effect is more likely to reflect the multifactorial and context-dependent nature of sleep health, rather than indicating that sleep is unimportant in the development of work burnout.

Given that the total mediating effect accounted for 42.36% and that all three routes were significant, the chain mediating model in the young group was consistent with the overall findings, according to subgroup analysis by age. This pathway was significant in the young group but not in the middle-aged group, where sleep health did not demonstrate significant mediating effects between work burnout and narcissistic rivalry. According to a study involving more than 150,000 American adults, young adults are the group most affected by sleep disorders (Grandner et al., 2012), and employment is a significant socioeconomic driver of sleep (Grandner, 2022). This may be partly related to the career development stage of younger adults. As they are still in the process of adapting to the workplace, their career development may be characterized by greater uncertainty, and concerns about “success” and “failure” may be more salient (Jin et al., 2023). In this context, younger employees in performance-oriented climates may be more likely to engage in overtime work to maintain competitiveness and obtain recognition and resources (Niu and Yang, 2022). Sustained overtime may also be associated with a higher risk of burnout, a possibility supported by Chen et al. (2025), who reported in a four-year longitudinal observational study of healthcare workers that prolonged overtime was associated with a higher risk of burnout. The mediating effect within the middle-aged group exhibited significant disparities. The overall mediating effect was 40.76%, which was marginally lower than that of the young group, and the Ind2 route was not statistically significant. The sleep health of middle-aged people may be more strongly influenced by physiological factors, such as alterations in sleep phases that result in shallower and easily disrupted sleep (Ohayon et al., 2004). In addition, melatonin secretion decreases with age (Verma et al., 2023), thereby attenuating the direct correlation between sleep and narcissistic rivalry. The effect value of the Ind1 path in the middle-aged group surpasses that of the young group, whereas the Ind3 path remains significant, suggesting that perceived stress may play a more prominent indirect role in this age group. This pattern may be related to the greater family and career pressures often experienced by middle-aged individuals. Narcissistic rivalry in this age group may be more closely linked to work burnout through perceived stress-related processes, which may in turn be linked to work burnout through sleep-related processes. Furthermore, a regression analysis revealed that in the middle-aged group, the positive associations of narcissistic rivalry and perceived stress with work burnout appeared stronger than those observed in the young group, and the negative predictive effect of sleep health is also more significant. These findings may suggest that stress and sleep-related difficulties are more strongly associated with work burnout among middle-aged individuals. This situation may be related to the decline in energy reserves and reduced coping resources among middle-aged people, which makes it more difficult for them to maintain stable work performance when facing the psychological impact of narcissistic rivalry. These age-specific patterns may also have important implications for organizational management, suggesting that intervention strategies should be tailored to employees at different life stages. Organizational managers should pay attention to the potentially adverse effects of narcissistic rivalry on employees’ perceived stress and work burnout, and may reduce these negative effects by implementing stress management training, fostering a healthy competitive culture, and providing interventions for interpersonal conflict. For the younger group, priority should be given to improving sleep health, reducing excessive social comparison and narcissistic rivalry through cognitive restructuring, and combining these efforts with stress management training. At the organizational level, burnout arising from unstable career development and intense interpersonal competition may be alleviated by regulating overtime, promoting healthy sleep routines, and creating a supportive workplace environment. For the middle-aged group, greater emphasis should be placed on reducing stress at its source by optimizing role expectations and promoting work–family balance. Organizations should also seek to reduce performance pressure and acknowledge the experience and contributions of middle-aged employees in order to alleviate burnout associated with role overload and psychological distress.

## 5 Limitations and future directions

This study has several limitations. First, the data were derived from self-administered questionnaires, and future research could combine interview and experimental methods to further improve the objectivity and reliability of the findings. Second, because this study employed a cross-sectional design, causal relationships among the variables cannot be established, and the conclusions therefore need to be further verified through longitudinal follow-up studies. At the same time, participants were recruited through convenience sampling within a stratified quota framework, which may have introduced selection bias and limited the external validity and generalizability of the findings. Future studies may consider using probability sampling methods to improve sample representativeness and enhance the applicability of the findings across a wider range of industries and regions. Third, this study only examined the mediating roles of perceived stress and sleep health, and the roles of other potential mediators or moderators in explaining how narcissistic rivalry influences employee burnout still warrant further investigation. In addition, only demographic variables, including gender, income, and education, were included as controls. Because the available dataset did not provide more detailed information, important work-related confounding factors such as job demands, organizational support, and working hours could not be taken into account (Christiansen et al., 2024). Future research should therefore incorporate a more comprehensive set of organizational and work-contextual variables to improve the explanatory power of the model and the accuracy of the findings. Furthermore, the brief SHS measure may have simplified the complex construct of sleep health to some extent. Subsequent studies could adopt more comprehensive multidimensional sleep measures and provide stronger theoretical and methodological justification for the operationalization of relevant variables.

## 6 Conclusion

The study revealed that the employed population in mainland China exhibits moderate levels of narcissistic rivalry, perceived stress, sleep health, and work burnout, with sleep health showing a significant negative correlation with the other three variables. Further analyses suggested that narcissistic rivalry was directly associated with work burnout and also showed indirect associations involving perceived stress and sleep health. These findings may provide a reference for potential intervention directions. Future intervention efforts may focus on the following three areas. First, individuals with high levels of narcissistic rivalry can be guided to adjust their competitive tendencies and reshape their self-cognition. Second, help with the establishment of effective stress regulatory mechanisms can be provided. Third, improving sleep quality should be an area of focus. Future research could explore the application of cognitive-behavioral therapy in this context or attempt to integrate personality interventions, stress management, and sleep enhancement strategies to improve intervention effectiveness.

## Data Availability

The raw data supporting the conclusions of this article will be made available by the authors without undue reservation.
